# The Issue of “Smart Drugs” on the Example of Modafinil: Toxicological Analysis of Evidences and Biological Samples

**DOI:** 10.3390/jox15010015

**Published:** 2025-01-17

**Authors:** Karolina Nowak, Agnieszka Chłopaś-Konowałek, Paweł Szpot, Marcin Zawadzki

**Affiliations:** 1 Department of Pharmacology, Faculty of Medicine, University of Opole, 48 Oleska Street, 45052 Opole, Poland; karolina.nowak@uni.opole.pl; 2 Department of Forensic Medicine, Division of Molecular Techniques, Wroclaw Medical University, Sklodowskiej-Curie 52, 50369 Wroclaw, Poland; agnieszka.chlopas-konowalek@umw.edu.pl; 3 Department of Forensic Medicine, Faculty of Medicine, Wroclaw Medical University, 4 J. Mikulicza-Radeckiego Street, 50345 Wroclaw, Poland; pawel.szpot@umw.edu.pl; 4 Department of Social Sciences and Infectious Diseases, Faculty of Medicine, Wroclaw University of Science and Technology, 27 Wybrzeze Wyspianskiego Street, 50370 Wroclaw, Poland; 5 Institute of Toxicology Research, 45 Kasztanowa Street, 55093 Borowa, Poland

**Keywords:** modafinil, evidence, biological material, forensic toxicology, UHPLC-QqQ-MS/MS

## Abstract

Cognitive enhancement through stimulants such as modafinil is becoming increasingly popular, with many individuals using prescription stimulants for non-medical purposes to improve alertness, attention, and mood. The misuse of such substances has raised concerns, particularly in forensic toxicology. The UHPLC-QqQ-MS/MS method was developed to quantify modafinil in evidentiary samples and biological materials. Additionally, the authors noted the presence of sodium adducts during the analysis of samples with high concentrations of modafinil. The method was validated for accuracy, precision, and linearity, with a concentration range of 0.1–10.0 µg/mL for the evidences and 1.0–100.0 ng/mL for blood. The method successfully detected modafinil as the sole substance in all evidences, with concentrations ranging from 90.7 to 120.8 mg, corresponding to 45.5% to 80.5% of the labeled dose. The method was applied to real post-mortem human cases, where, among others, the concentration of modafinil in blood was 110 ng/mL, whereas, in another case, the concentration of modafinil in the putrefaction fluid exceeded 1000 ng/mL. The developed UHPLC-QqQ-MS/MS method is effective for the quantification of modafinil in evidentiary samples and biological materials, offering a reliable tool for forensic toxicology applications. This method can be used to evaluate modafinil use in both legal and illicit contexts, including cases of overdose or misuse.

## 1. Introduction

Some people attempt to enhance their cognitive functions, such as alertness, attention, concentration, and memory, as well as psychological functions, such as mood and sleep [1,2,3], through the use of stimulants [4,5]. This phenomenon is referred to as pharmaceutical neuroprotective enhancement or cognitive enhancement (CE) [1,6]. Substances used for CE include over-the-counter products, such as caffeine pills and energy drinks [3,7], and prescription stimulants, like methylphenidate and modafinil, which, despite their therapeutic effects [1], are also misused without medical discretion as so-called “smart drugs” [8,9,10,11,12].

Approximately 0.5 million young people [13,14] and 4.8 million adults (including 2.5 million young adults aged 18–25) [15,16,17] reported using stimulants for non-medical purposes in 2015. Among American adults, approximately 6.6% used prescription stimulants (for both therapeutic and “smart drug” uses), with 4.5% using them as prescribed without misuse and 2.1% misusing prescription stimulants at least once [9]. Epidemiological data indicate that 5–9% of primary and secondary school children and 5–35% of college-aged individuals in the United States used stimulants, primarily for cognitive enhancement but also to experience a “high” [12,18,19,20]. In the adult population (among students and academic professionals) in the U.S., the most common reasons for non-medical stimulant use include performance enhancement (e.g., increased alertness, concentration, productivity, study aid, and motivation to complete tasks—reported by 78% of respondents). Additionally, 15.5% cited experimentation or seeking a “high” as motivation for misusing prescribed stimulants, while about 4% reported weight loss as the primary motivation for non-medical stimulant use [9,21].

Among German students, 1.55% used prescribed stimulants, and 2.6% used illegal stimulants to enhance cognitive performance [11]. In 2010, 34% of Swiss employees reported experiencing chronic stress compared to only 7% of participants in a similar study in 2000 who reported frequent high levels of stress [22]. Approximately 6% of Swiss employees took substances to enhance cognitive function, and 15% used substances to relax and unwind after stressful workdays [23]. In a study among French medical students, 0.8% admitted to using modafinil to improve academic performance, concentration, and alertness [24]. In an anonymous survey among surgeons, 2.2% of respondents (*n* = 957) reported using modafinil [25]. Other subjective benefits of using cognitive-enhancing drugs include increased mental endurance [26] and greater interest in work [27]. Reported reasons for using cognitive enhancers include anxiety about academic failure, the need to meet high professional demands, overcoming procrastination, and boosting motivation [28].

Modafinil (MOD; 2-benzhydrylsulfinylacetamide) is considered the most commonly used over-the-counter cognitive enhancer [1,29]. It is a centrally acting sympathomimetic drug and a non-amphetamine stimulant, similar to methylphenidate and piracetam, exhibiting a complex neurochemical profile that affects both online and offline processes. MOD is currently used in the treatment of narcolepsy [27,28,29,30] and idiopathic hypersomnia, demonstrating significant therapeutic benefits by promoting wakefulness and alertness in shift work disorder [1,31] and obstructive sleep apnea [30,32,33,34]. It also showed potential in treating neurodegenerative disorders (e.g., Alzheimer’s disease) and cognitive impairment in schizophrenia [35]. MOD may enhance cognitive function in healthy individuals [36] and was noted to improve symptoms in children and adults with ADHD [37]. Additionally, modafinil has gained attention for its potential in treating cocaine addiction [38] and methamphetamine addiction [39].

Modafinil was first introduced to the pharmaceutical market in France in the 1990s as an eugeroic drug for narcolepsy. In 1998, the Food and Drug Administration (FDA) in several countries (outside Europe) approved MOD for the treatment of narcolepsy, and in 2003, for shift work sleep disorder and obstructive sleep apnea [40].

The mechanism of action of MOD is complex and not fully understood [41]. Although modafinil is classified as a psychostimulant, it operates along a neural pathway distinct from that of amphetamine and cocaine [33]. It was shown to directly or indirectly activate the dopaminergic [42,43], glutamatergic [44], noradrenergic [30,42], and serotonergic [42] systems in various brain regions, including the prefrontal cortex, hippocampus, hypothalamus, and striatum, while inhibiting GABAergic pathways in these same regions [1,8,21,30,36,41,44,45,46]. However, Volkow et al. demonstrated the addictive potential of modafinil through the inhibition of dopamine reuptake by DAT in the nucleus accumbens [43].

After oral administration, MOD is rapidly absorbed from the gastrointestinal tract. The therapeutic dose for adults ranges from 200 to 400 mg per day, administered in two divided oral doses [43,47]. A 600 mg dose (administered orally) in humans did not produce any patient-reported psychoactive effects (such as a high, rush, or stimulation) [48]. Even the intake of modafinil by adults in doses several times higher than the recommended daily doses does not cause life-threatening effects. Modafinil can be abused due to its stimulant effects and can cause aggressive states or psychotic pathologies, which requires increased monitoring. The uncontrolled consumption of this compound can also cause agitation, insomnia, restlessness, irritability, aggression, confusion, nervousness, tremors, and palpitations [49,50,51,52]. No life-threatening toxicity occurred, and to date, no fatal overdoses occurred involving modafinil alone, but the simultaneous overdoses of multiple drugs, including modafinil, have led to death [53]. The literature reported a case of a 15-year-old girl who took 5000 mg (5 g) of modafinil at one time for suicidal purposes. Despite taking 60 times the recommended dose of modafinil, this did not result in death [51]. Another case involved a 17 year old who ingested 12 g of modafinil and required hospitalization due to altered consciousness and encephalopathy caused by intoxication. Following discharge, the patient exhibited persistent psychotic symptoms, including visual and auditory hallucinations [50]. The data on its use in toxic doses are also limited.

Following a single oral dose of 100 mg modafinil in 12 children, the average maximum plasma concentration was reached in approximately 2.1 h, averaging 2.2 mg/L. A single 200 mg oral dose administered to 24 adult patients reached a mean peak plasma concentration of 4.8 mg/L in about 1.8 h [54]. The oral bioavailability of MOD is 40–60%. Intravenous administration in humans was excluded due to its low solubility in water [55].

MOD is available in oral tablets [47]. It consists of R-(−) and S-(+) enantiomers and was originally prescribed as a racemate under trade names such as Provigil^®^, Modasomil^®^, Modavigil^®^, Modiodal^®^, Modalert^®^, and Vigil^®^ [56]. The S- and R-isomers have equal pharmacological activity but differ in pharmacokinetics. The R-isomer, marketed as armodafinil, has a half-life of approximately 10–14 h, whereas the *S*-isomer is eliminated more rapidly with a half-life of 3–5 h [57]. In individuals with liver or kidney impairment and in older adults, the half-life may be prolonged. In patients with liver cirrhosis, the elimination of modafinil decreases by approximately 60% [58].

Modafinil binds moderately to plasma proteins (approximately 60%), primarily to albumin, which suggests a low risk of interactions with drugs that are strongly protein-bound. The drug is metabolized in the liver through the cytochrome P450, primarily by CYP3A4 and, to a lesser extent, by CYP2C9. Additionally, armodafinil is both a substrate and an inhibitor of P-glycoprotein [59]. Moreover, MOD has the potential to inhibit CYP2C19. Therefore, the co-administration of modafinil with drugs such as diazepam, phenytoin, and propranolol may increase the blood levels caused by these medications. In patients receiving these drugs, dose adjustments may also be necessary [60]. Approximately 10% of the administered dose is excreted as unchanged in the urine, while around 40–60% undergoes conjugation to modafinilic acid, which, along with hydroxy-modafinil and modafinil sulfone, is pharmacologically inactive [54,55].

The objective of this study was to develop and validate the UHPLC-QqQ-MS/MS method for the simultaneous detection of modafinil in samples (tablets) and its quantification in biological materials, such as peripheral blood, urine, vitreous humor, putrefaction fluid, liver, kidney, and brain. The validated methods were applied to analyze five samples in the form of tablets containing modafinil, purchased online, as well as for the determination of modafinil concentrations in post-mortem biological materials from three cases (unrelated to modafinil intoxication).

## 2. Materials and Methods

### 2.1. Chemicals

Water (chemsolve^®^ LC–MS), acetonitrile (chemsolve^®^ LC–MS), and methanol (chemsolve^®^ LC–MS) were purchased from WITKO (Łódź, Poland); formic acid and ammonium formate were purchased from Chem-Lab NV (Zedelgem, Belgium); modafinil was purchased from Supelco (Roun Dock, TX, USA), internal standard methylphenidate-*d*_9_ was purchased from Ceriliant (Round Rock, TX, USA). Structures of modafinil and IS are presented in Figure S1 in Supplementary Materials.

### 2.2. Tested Samples

**Figure 1:**
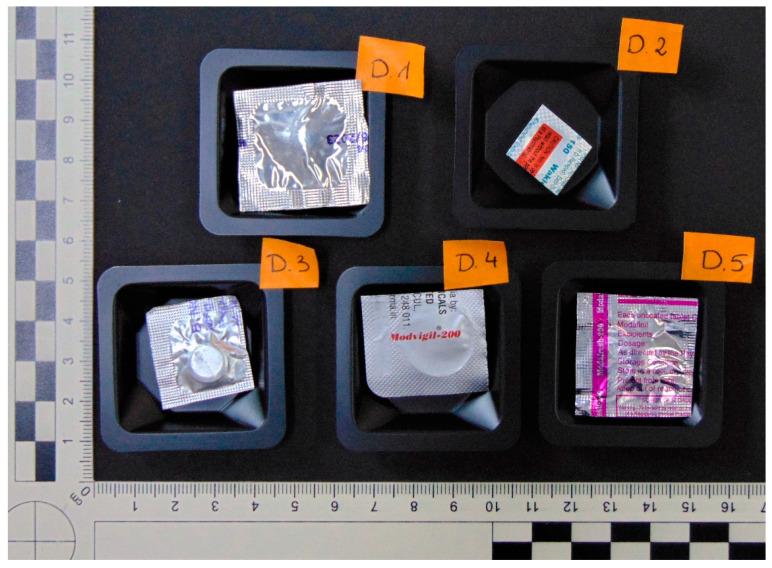

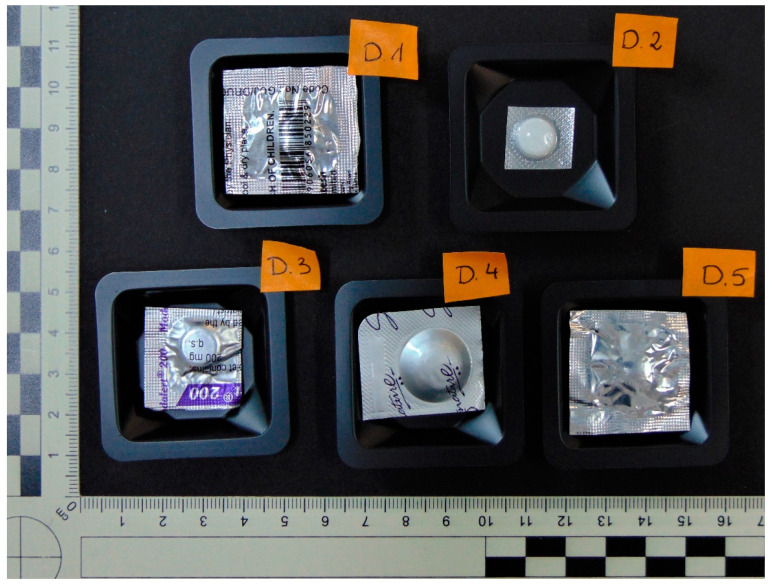
Fragments of blisters with tested samples. View from two sides of blisters.

Five evidentiary samples (designated as D1–D5) were analyzed. The samples were obtained from volunteers professionally engaged in emergency medical services, who reported purchasing the products from websites. The products were originally claimed to have originated from Asian countries. Each specimen was encased within a fragment of a blister, as illustrated in Figure 1 (D1—unknown name, D2—“Waklert^®^ 150”, D3—“Modalert^®^ 200”, D4—“Modvigil^®^-200”, D5—“Modafresh-200”). The blister packs were made of high-quality materials and, together with the printed labels, created the impression of authentic products. The individual blisters were enveloped in plastic bags, with handwritten notes. Notably, each tablet exhibits a circular, convex shape and a lustrous, snow-white appearance (Figure S2 in Supplementary Materials).

#### 2.2.1. Working Solutions, Calibration Curve, and Quality Control Samples

The standard solution (at concentration of 100 µg/mL) of modafinil was prepared in acetonitrile, and the internal standard (IS) was prepared in methanol. The working solutions of different concentrations were prepared by the dilution of the standard solution with acetonitrile and were stored with stock solutions at −20 °C.

##### Evidentiary Samples

Calibration points (*n* = 9; 0.1, 0.2, 0.5, 0.8, 1.0, 2.0, 5.0, 8.0, and 10.0 µg/mL) and quality control (QC) samples (*n* = 3) were prepared by spiking the appropriate working solution into acetonitrile solutions.

##### Biological Materials

Calibration points (*n* = 7; 1, 2.5, 5, 10, 25, 50, and 100 ng/mL) and QC samples (*n* = 2) were prepared as outlined above.

#### 2.2.2. Procedure

##### Evidentiary Samples

Each tablet was weighted on an analytical scale (AS 220.R2, RADWAG, Radom, Poland) and then transferred into 10 mL plastic vials with 5 mL of methanol (LC-MS grade), tightly closed, and sonificated for 30 min (Ultrasonic Cleaner USC300TH, VWR, Gdańsk, Poland). Next, the samples were centrifuged at 2540× *g* at 4 °C for 10 min. The supernatant was diluted with methanol by 10,000 folds. Then, 10 μL of the diluted sample was transferred into a 2 mL Eppendorf tube containing 10 μL of IS (methylphenidate-*d*_9_, 1 μg/mL) and 80 μL of methanol. After mixing, the solution was transferred into inserts for autosampler vials and analyzed by ultra-high performance liquid chromatography–triple quadrupole tandem mass spectrometry (UHPLC-QqQ-MS/MS). The injection volume was 2 μL. All samples were prepared and analyzed in duplicates.

##### Biological Materials

Post-mortem samples of various biological matrices were designated for routine toxicological analysis: peripheral blood, urine, vitreous humor, putrefaction fluid, fragments of liver, kidney, and brain. Samples of post-mortem biological fluids (0.2 mL each) and homogenates of organs (0.2 g of each homogenate) were prepared according to the standard procedure of our laboratory (liquid–liquid extraction (LLE) with 2 mL ethyl acetate, pH = 9 after the addition of 0.2 mL of ammonium carbonate and 0.02 mL of IS (methylphenidate-*d*_9_ in concentration of 1 µg/mL), vortexed for 10 min, and centrifuged for 10 min at 2540× *g* at 4 °C; the supernatant evaporated to dryness under the stream of N_2_, and, finally, the dry residues diluted with 0.05 mL of methanol) [61,62,63,64,65]. This same procedure was used to perform the calibration curve and method validation. Due to concentrations in biological materials exceeding the ULOQ, additional measurements were performed following prior dilution. The dilution effect was also assessed as part of the method validation.

#### 2.2.3. Chromatographic and Spectrometric Conditions

Qualitative and quantitative toxicological analyses were performed using an ultra-high-performance liquid chromatograph (Nexera X2, Shimadzu, Kyoto, Japan). The separation was completed on Kinetex XB-C18 2.6 μm 2.1 × 150 mm column (Phenomenex, Torrance, CA, USA). The thermostat was set at 40 °C. The mobile phase included (A) 10 mM ammonium formate in water with 0.1% formic acid and (B) acetonitrile with 0.1% formic acid. The gradient elution was carried out at a constant flow of 0.4 mL/min. The applied gradient was as follows: 0 min, 5% B; 12 min, 98% B; 14 min, 98% B; and 15 min, 5% B; and those conditions were carried out for 5 min. This mobile phase composition was employed for the quantitative determination of modafinil in evidences (tablets) and biological materials.

In addition, in order to check the influence of the composition of the mobile phase on the ability to monitor the modafinil precursor as 274 *m*/*z* ([M + H]^+^), beyond the phase used for the quantitative analysis, we performed two additional experiments. In the first one, the phase composition was pure water (A) and pure acetonitrile (B), while, in the second one, we used water with 0.1% formic acid and acetonitrile with 0.1% formic acid (B). In both cases, the gradient elution remained the same.

A triple quadrupole mass spectrometer (LCMS-8050, Shimadzu, Kyoto, Japan) equipped with an electrospray ionization (ESI) source was used for the detection of the investigated compounds. Analyses were performed in two directions: the 1st was general untargeted screening (scan mode, range of 50–1000 *m*/*z*) for checking for any impurities, and the 2nd was the determination of the investigated substances in the multiple reaction monitoring (MRM) positive mode.

The following MS parameters were fixed for both targeted (MRM) and untargeted analyses (scan mode): nebulizing gas flow, 3 L/min; heating gas flow, 10 L/min; interface temperature, 250 °C; desolvation line (DL) temperature, 200 °C; heat block temperature, 350 °C; and drying gas flow, 10 L/min. A summary of precursor and product ions, collision energies, dwell time, Q1–Q3 pre-bias voltages, and retention time for modafinil and IS are presented in Table 1.

#### 2.2.4. Validation

##### Evidentiary Samples

The validation did not account for tablet morphology or the stability of the substance in evidence samples. Validation included the determination of the following parameters: limit of detection (LOD), lower limit of quantification (LLOQ), upper limit of quantification (ULOQ), linearity, coefficient of determination (*R*^2^), intra- and inter-day precision, and intra- and inter-day accuracy (*n* = 5; concentration: 0.2, 1.0, 8.0 µg/mL). Additionally, to verify the selectivity of the method, samples in the form of tablets containing quetiapine were prepared and analyzed using the presented method (applying the same dilutions for the evidence samples).

The method validation was based on the recommendations outlined in the Guidance for the Validation of Analytical Methodology and Calibration of Equipment used for Testing of Illicit Drugs in Seized Materials and Biological Specimens (United Nations Office on Drugs and Crime, UNODC) [66].

##### Biological Materials

Validation included: LOD, LOQ, ULOQ, *R*^2^, intra- and inter-day precision, intra- and inter-day accuracy, recovery, and matrix effect (*n* = 5; concentration: 10 and 100 ng/mL). Method validation was performed in accordance with the Scientific Working Group for Forensic Toxicology (SWGTOX) standard practices for method validation in forensic toxicology [67]. To verify the selectivity of the method, blank samples of the analyzed matrices were routinely tested. For matrices such as vitreous humor and organ fragments, biological materials collected from deceased individuals were analyzed. These materials, previously examined and designated for disposal per the experimenter’s decision, were used for this purpose.

## 3. Results

### 3.1. Evidentiary Samples

**Figure 2:**
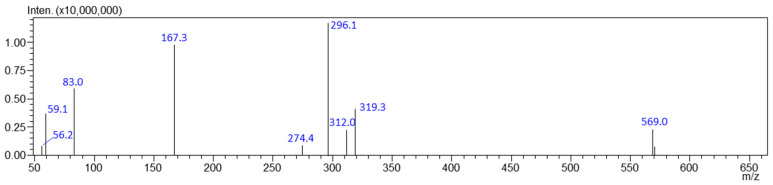
Q3 scan of one of analyzed evidences.

**Figure 3:**
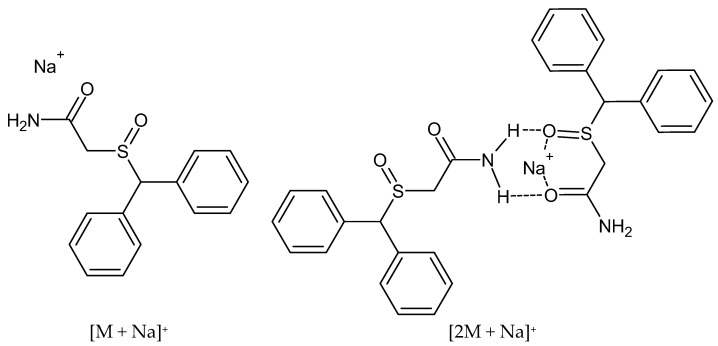
Structures of sodium adduct that can be observed in analyzed evidences.

Figure 2 shows the characteristic precursor ions corresponding to modafinil or its sodium adducts, observable in the positive Q3 scan mode of evidentiary samples (adducts were observed for the analytical standard at high concentrations). Figure 3 presents the structures of two sodium adducts: [M + Na]^+^ (296 *m*/*z*) and the dimer [2M + Na]^+^ (569 *m*/*z*). Figure S3 in the Supplementary Materials illustrates the proposed fragmentation of the 296 *m*/*z* ion.

**Figure 4:**
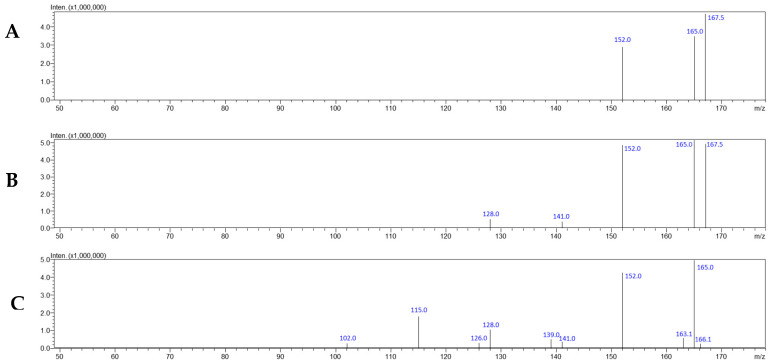
Mass spectra of modafinil in product ion scan mode (CE: 10 (**A**), 20 (**B**), and 35 V (**C**)).

Figure 4 shows product ion scan mass spectra of modafinil (precursor ion set to 167 *m*/*z*). Figure S4 (in Supplementary Materials) shows chromatograms with MRM transitions typical for modafinil and IS.

The linear concentration range was 0.1–10.0 µg/mL, LOD was 0.05 µg/mL, LLOQ was 0.1 µg/mL, ULOQ was 10.0 µg/mL, and *R*^2^ > 0.9999. Intra- and inter-day precision, as well as intra- and inter-day accuracy, for all of three concentrations (low-QC, medium-QC, high-QC; *n* = 5) were below 5% (detailed information in Table S1 in Supplementary Materials). The method was successfully applied to quantify modafinil in the evidence samples. In all cases, modafinil was the only active substance detected (filling agents were not analyzed). The modafinil content in individual samples ranged from 90.7 to 120.8 mg, corresponding to 45.5% to 80.5% of the stated dose, as indicated on the packaging. Detailed results are presented in Table 2.

### 3.2. Biological Materials

The linear concentration range was 1.0–100 ng/mL, LOD was 0.5 ng/mL, LLOQ was 1.0 ng/mL, ULOQ was 100 ng/mL, and *R*^2^ > 0.9999. The remaining results of the method validation is shown in Table 3.

Similarly to the evidentiary samples, the method was successfully applied to determine the MOD in biological materials. Table 4 shows the concentrations of MOD in three post-mortem cases. Additionally, in case 1, modafinil sulfone was qualitatively detected in all tested samples.

## 4. Discussion

Modafinil, in addition to its approved use for treating excessive sleepiness—classified as a “smart drug”—is frequently misused by healthy individuals, including night-shift workers, students, and healthcare professionals [8,9,10,11,12]. These individuals use modafinil to combat fatigue and drowsiness, enhance concentration and alertness, improve memory and cognitive abilities, and cope with stress and anxiety; some also misuse it to experience a euphoric “high” [12,18,19,20].

Although the long-term effects of modafinil use in healthy individuals remain unknown, the drug is easily accessible online, often with limited information about its use and potential harmful effects [68,69]. Other acquisition options include online shops, retail outlets, pharmacies (with or without a prescription), and sources such as colleagues, friends, or family [68]. The core issue lies in the uncertainty regarding whether the purchased product is an authentic medicinal product, with its qualitative and quantitative composition matching the approved characteristics, or if it deviates from the manufacturer’s guaranteed standards. Products purchased from the black market, which often closely mimic the appearance of legitimate pharmaceutical products, may pose serious risks to the health and safety of users. This could inspire trust among users unaware of the potential risks, leading them to mistakenly believe they are dealing with a product of controlled quality. These risks particularly arise from discrepancies in the actual content of the active substance in such products.

In cases of high modafinil concentrations in evidentiary samples, both precursor ions and sodium adducts, such as [M + Na]^+^ and the dimer [2M + Na]^+^, can be observed. In our study, these adducts formed independently of the mobile phase compositions we tested. Pan [70] suggested that dimer formation can be concentration-dependent, indicating a non-covalent association, with the abundance of dimer ions correlating with the expected number of hydrogen bonds within the molecules. Additionally, Grocholska et al. [71] noted that the greatest challenge in forming non-covalent complexes in the ESI-MS mode unequivocally proved that the binding is specific. Mass spectrometry often detects nonspecific complexes, influenced by ionization source parameters and analyte concentrations.

In biological samples, the 274 *m*/*z* ion is typically not observed, with only diphenylmethylium being detected. The absence of the [M + H]^+^ ion is attributed to its low stability in the ion source, which promotes the formation of diphenylmethylium via a charge-directed mechanism [72].

Table S2 in the Supplementary Materials presents the selected parameters for modafinil detection methods using liquid chromatography coupled with an MS/MS detector in biological samples. To date, LC-MS/MS techniques were not used for the quantitative analysis of modafinil in evidentiary samples. Our developed method was applied to both the samples and biological materials.

For the samples we analyzed, modafinil was the only detected substance. Its concentration in the individual evidentiary samples ranged from 90.7 to 120.8 mg, corresponding to 45.5 to 80.5% of the labeled dose (based on blister pack information). Assi et al. [73] analyzed eight tablets purchased from four different websites, with labeled doses of 100 and 200 mg of modafinil. The modafinil content in these tablets ranged from 57.6 to 72.7%. The researchers employed the following methods to determine the content of MOD: Fourier transform-infrared (FTIR), near-infrared (NIR), and Raman spectroscopy. Harvanová and Gondová [74] conducted enantioseparation studies on *R*- and *S*-modafinil in five samples: three were commercial pharmaceutical preparations of modafinil and armodafinil purchased from a pharmacy and an online pharmacy, while the other two were illegal modafinil powders bought online from China. The relative standard deviation (RSD) for the three pharmacy-obtained samples ranged from 0.3 to 0.7%, while the modafinil content in the illegal powders was 99.5% and 96.9%, respectively (the sum of *S*- and *R*-MOD). The authors analyzed, among others, Modvigil™ (200 mg) and Waklert^®^ (150 mg), obtained from a local drug store in Ukraine and an online pharmacy (also in Ukraine). The content of *R,S*-MOD in Modvigil™ was 198.4 ± 0.5 mg (out of the declared 200 mg), while the content of *R*-MOD in the Waklert^®^ product was 150.8 ± 0.5 mg (out of the declared 150 mg). In contrast, in our study, products with the same names contained MOD at levels of 80.5% (Waklert^®^) and 54.8% (Modvigil^®^), respectively. The samples we examined most likely came from the black market. In addition, we did not know the expiration date, which is also crucial in the case of an active substance content. This is a clear example demonstrating that counterfeit products, mimicking pharmaceutical-grade items, fall outside quality control standards and have compositions (and contents) that differ from those declared on the packaging. The authors [74] employed an HPLC-UV method for the determination (calibration curves in a range of 5–150 µg/mL; *R*^2^ > 0.999; LOD: 15 (*R*-MOD) and 20 ng/mL (*S*-MOD); LLOQ: 45 (*R*-MOD) and 60 ng/mL (*S*-MOD); recoveries: 100.5–102.3% (SD from 0.4 to 1.0% for both enantiomers)).

Among the methods developed to date for the determination of modafinil in biological samples, and in pharmaceutical formulations, it is worth mentioning those utilizing e.g., high-performance liquid chromatography with ultraviolet detection (HPLC-UV) [75], mass spectrometry (MS) [76], gas chromatography coupled with mass spectrometry (GC-MS) [77], and capillary electrophoresis with PDA detection (CE-PDA) [78,79] methods were developed.

Table S2 in the Supplementary Materials presents selected parameters of LC-MS/MS methods for the determination of modafinil in biological materials. The extraction methods include LLE, solid-phase extraction (SPE), and protein precipitation (PP). All methods listed in Table S2 utilized a C18 column, a commonly used type of column for analyses in clinical and forensic toxicology. The composition of the mobile phase in most methods was a mixture of water and acetonitrile, with or without additives such as acetic acid and ammonium formate. Similarly, both our method and the majority of methods listed in Table S2 for modafinil determination employed a gradient program for the mobile phase.

In our method, the injection volume was among the smallest, and some methods used non-deuterated analytical standards as internal standards, which would render such methods unsuitable for forensic toxicology. The LLOQ in our method was 1 ng/mL, comparable to other methods for determining modafinil in plasma samples. For urine samples, LC-MS/MS methods typically have higher LOD and LLOQ values, in the range of 100–300 ng/mL. The recovery rates, both in our method and in other methods described in Table S2, were satisfactory.

Modafinil is available by prescription and is characterized by low potency, requiring high doses to achieve significant stimulant effects [80]. Its onset of action is slow, typically occurring 60–100 min after administration [81].

Overdoses of modafinil are sporadic but can result in life-threatening toxicity [68,69]. One reported case involved a healthy 32-year-old man who was found unconscious, face down on the bathroom floor, with evidence of vomiting. An empty blister pack of modafinil and equipment for insufflation were discovered nearby. The patient was admitted to the emergency department, where he was intubated while sedated and then transferred to the intensive care unit (ICU). Upon regaining lucidity, the patient confirmed having insufflated an estimate of over 600 mg and ingesting a methylphenidate (less than 200 mg). Clinicians attributed his condition to severe hyponatremia and subsequent cerebral edema caused by the modafinil overdose [53].

In general, modafinil overdose symptoms are mild and include headaches, dizziness, nausea, tachycardia, insomnia, nervousness, diarrhea, anxiety, and abdominal pain [58]. Clinical studies showed that doses up to 1200 mg/day for 7–21 days or a single dose as high as 4500 mg do not typically result in life-threatening effects. However, other adverse effects were observed, including anxiety, irritability, aggression, confusion, tremors, palpitations, chest pain, and sleep disturbances. Doses exceeding 1000 mg were associated with dyskinesia, likely due to dopaminergic activity [51].

Accidental poisonings involving modafinil were also reported in children. The highest reported accidental ingestion involved a 3-year-old boy who consumed 800–1000 mg (50–63 mg/kg) of modafinil but remained in a stable condition [51]. In two cases involving teenage girls, symptoms included tachycardia, insomnia, agitation, dizziness, and anxiety, with serum modafinil concentrations of 13–18 mg/L measured 18–24 h post-hospitalization [54].

In another instance, a 14-year-old girl ingested 20 tablets containing 200 mg modafinil each and 10 tablets containing 10 mg escitalopram each [82]. Her symptoms were similar to those previously described [58], and her serum modafinil concentration was measured at 18 mg/L 24 h after hospital admission [82]. A separate case involved a 16-year-old girl who presented with rapid heartbeat, headache, “tingling sensations”, transient chest pain, and behavioral changes, including anxiety and visual hallucinations. A serum sample taken 18 h post-admission revealed a modafinil concentration of 13 mg/L, although the timing and amount of modafinil ingested were unclear [83].

Modafinil is often regarded as having a very low potential for abuse and dependence. Many believe it is not addictive because it does not directly target the brain’s reward circuitry [84]. However, several cases of modafinil addiction were reported. Four case reports documented modafinil dependence syndrome [85], involving individuals with pre-existing psychiatric disorders, such as schizoaffective disorder [52,85] and bipolar disorder [86], as well as a history of methamphetamine dependence [87]. Notably, no cases of abuse or dependence were reported in patients taking modafinil for approved medical conditions.

Modafinil should be used with caution in patients with a history of psychiatric disorders, such as psychosis, depression, manic excitation, major anxiety disorders, psychomotor agitation, insomnia, and substance abuse [47].

Furthermore, there is limited literature on blood concentrations of modafinil in cases involving DUID (driving under the influence of drugs), suspected misuse, and/or intoxications.

One study presented five cases encountered during routine work, highlighting various scenarios where modafinil ingestion was confirmed [47]. These included three cases of DUID (2.1–3.8 mg/L of modafinil in blood), one case of bodily harm (1.3 mg/L), and one case later confirmed as intoxication (approx. 34 mg/L of modafinil). The patient in the final case experienced loss of consciousness, somnolence, disorientation, and slowed responses. A headache, a known side effect of modafinil, was reported the following day when the patient regained full consciousness.

According to Baselt, the therapeutic range of modafinil concentrations in plasma is 2–9 mg/L [54], while Regenthal et al. reported a narrower therapeutic range of 0.9–3.3 mg/L [88]. In the cases described, the modafinil concentrations measured were below therapeutic levels and did not contribute to the individual’s death. For example, the blood concentration reported in case 1 of this study (110.0 ng/mL) was significantly lower than the concentrations observed in other cases [47]. This difference may be attributed to the fact that none of the reported cases involved intentional self-harm using modafinil. It may also be related to the use of black-market modafinil, which, as demonstrated in our research, may contain a lower dose of the drug than stated on the packaging. Moreover, it is difficult to interpret concentrations in cases where modafinil is detected in putrefaction fluids or solid tissues (e.g., our case 2). In addition, modafinil was not the only substance identified in all three cases. Among the substances detected were THC with metabolites, CBD, cocaine with metabolites, amphetamine, methamphetamine, MDMA, MDA, ketamine with metabolite, 2C-B, 4-chloromethcathinone (4-CMC), mephedrone, tadalafil, mianserine with metabolite, levamisole, sildenafil with metabolites, aripiprazole with metabolite, and several substances from the benzodiazepine class.

Modafinil intake appears to be relatively uncommon in both forensic and clinical toxicology. According to literature data [47], police-related cases were all submitted to the laboratory in 2015, while the hospital sample was sent in 2013. The statistics indicate that approximately 14,000 clinical and forensic toxicology analyses were conducted annually. In 2016, no cases of modafinil intake were reported [47]. From the authors’ experience, after analyzing over 1,000 forensic toxicology samples annually, modafinil was detected in only three cases (two in 2023 and one in 2024). These findings should be interpreted as incidental and do not suggest an increasing trend in modafinil consumption.

It remains unclear whether this low number of reported abuse cases is due to the limited availability of the drug, or if it reflects the drug’s lower abuse potential. Another possible explanation is that patients abusing modafinil may not seek medical assistance or contact poison centers for consultation.

Additionally, forensic toxicology laboratories do not receive samples from all autopsies, as forensic pathologists often confirm causes of death unrelated to xenobiotic poisoning. Another important consideration is that routine diagnostic testing typically focuses on detecting ethanol, highly toxic drugs, and substances like benzodiazepines, barbiturates, and opioids. Standard toxicology screening panels often do not detect “smart drugs”, including modafinil.

## 5. Conclusions

The developed UHPLC-QqQ-MS/MS method was successfully applied for the quantitative analysis of modafinil in the evidentiary samples and biological matrices, making it suitable for use in forensic toxicology. The method was validated for accuracy, precision, and linearity, with a concentration range of 0.1–10.0 µg/mL for evidentiary samples and 1.0–100.0 ng/mL for blood. For the evidentiary samples, intra- and inter-day precision, as well as intra- and inter-day accuracy, for all of three checked QC concentrations were below 5%. For biological materials, intra- and inter-day precision, as well as intra- and inter-day accuracy, for both QC levels were below 15%, recovery was 111.1% for low QC and 110.8% for high QC, and the matrix effect was 105.8% for low QC and 110.2% for high QC samples. In the examined evidentiary samples, modafinil was the only detected active substance; however, its content was lower than the amount declared on the packaging, in some cases, by over 50%. Although modafinil was not the cause of death in the cases studied and was present at low concentrations (case 1: 110 ng/mL in blood, >3000 ng/mL in urine, 30 ng/mL in vitreous humor, 250 ng/g in liver, 210 ng/g in kidney, 410 ng/g in brain; case 2: >1000 ng/mL in putrefaction fluid; case 3: 14 ng/mL in urine), its presence indicates that “smart drugs” such as modafinil should be routinely monitored in toxicological investigations.

## Figures and Tables

**Table 1:** MRM conditions used in the UHPLC/ESI-MS/MS analysis of modafinil and IS.

Compounds	Precursor Ion [*m*/*z*]	Product Ion [*m*/*z*]	Dwell (msec)	Q1 Pre-Bias [V]	Collision Energy [V]	Q3 Pre-Bias [V]	Retention Time [min]
Modafinil	166.9	152.1 * 115.2	2.0	−11 −11	−22 −40	−15 −19	5.57
Methylphenidate-*d*_9_	242.9	93.1 * 61.1	1.0	−11 −15	−22 −49	−15 −10	4.42

* Ions selected for quantitative analysis.

**Table 2:** Results of toxicological analyzes of evidentiary samples.

No.	Determined Substance	Tablet Weight [mg]	Amount of Substance in the Sample [mg]	Percentage of the Total Weight of the Tablet [%]	Probable Dose (Data from the Bister) [mg]	Percentage of the Dose [%]	Presence of Impurity
D.1	Modafinil	401.0	90.7	22.5	200 *	45.5	No
D.2	Modafinil	269.2	120.8	44.9	150	80.5	No
D.3	Modafinil	318.4	92.0	28.9	200	46.0	No
D.4	Modafinil	333.0	109.7	32.9	200	54.8	No
D.5	Modafinil	341.1	105.3	30.8	200	52.6	No

* Information from handwritten inscription on the plastic bag.

**Table 3:** Results of biological material method’s validation; *n* = 5.

Validation Results		
Intra-day precision [%]	10 ng/mL	2.3
	100 ng/mL	14.9
Intra-day accuracy [%]	10 ng/mL	8.6
	100 ng/mL	5.8
Iner-day precision [%]	10 ng/mL	2.9
	100 ng/mL	10.0
Inter-day accuracy [%]	10 ng/mL	13.0
	100 ng/mL	6.3
Recovery [%]	10 ng/mL	111.1
	100 ng/mL	110.8
Matrix effect [%]	10 ng/mL	105.8
	100 ng/mL	110.2

**Table 4:** Modafinil concentrations in authentic forensic cases (biological fluids and tissues).

Concentrations of Modafinil [ng/mL ^a^ or ng/g ^b^]							
**Case Number/Sex**	**Biological Material**						
	**Blood**	**Urine**	**Vitreous Humor**	**Putrefaction Fluid**	**Liver**	**Kidney**	**Brain**
Case 1/Male	110	>3000	30	(−)	250	210	410
Case 2/Male	(−)	(−)	(−)	>1000	(−)	(−)	(−)
Case 3/Male	0	14	(−)	(−)	(−)	(−)	(−)

(−) material was not collected, ^a^ concentration for biological fluids, ^b^ concentration for solid tissues.

## Data Availability

The data that supports the findings in this study are available from the corresponding authors upon reasonable request.

## Supplementary Materials

The following supporting information can be downloaded at: https://www.mdpi.com/article/10.3390/jox15010015/s1. Figure S1: Structures of modafinil and IS (methylphenidate-*d*_9_). Figure S2: Tablets after removal from blisters. Figure S3: Proposed fragmentation of sodium adduct of modafinil (296 *m*/*z*). Figure S4: MRM of modafinil in blank methanolic solution sample (A), and stock solutions: 0.05 µg/mL (LOD) (B), 0.1 µg/mL (LLOQ) (C), 1.0 µg/mL (D), and 10.0 µg/mL (ULOQ) (E); MRM of IS methylphenidate-*d*_9_ in 1.0 µg/mL (F). Table S1: Results of evidentiary sample method’s validation; *n* = 5. Table S2: Comparison of selected parameters of methods for determining modafinil in biological matrices with the liquid chromatography with MS/MS detection system [89,90,91,92,93,94,95,96,97,98].
