# Supplementary material for: The Issue of “Smart Drugs” on the Example of Modafinil: Toxicological Analysis of Evidences and Biological Samples
Source: J Xenobiot. 2025 Jan 17;15(1):15. doi: 10.3390/jox15010015 (PMC11755661; doi:10.3390/jox15010015)
Supplement: Supplementary file 1 [file jox-15-00015-s001.zip › jox-3375146-supplementary.pdf]

SUPPLEMENTARY METERIALS

The Issue of "Smart Drugs" on the Example of Modafinil: Toxicological Analysis of Evidences and Biological Samples

Karolina Nowak [1,†], Agnieszka Chłopaś-Konowałek [2,†], Paweł Szpot [3], Marcin Zawadzki [4,5*]

[1] Department of Pharmacology, Faculty of Medicine, University of Opole, 48 Oleska Street, 45052 Opole, Poland; karolina.nowak@uni.opole.pl
[2] Department of Forensic Medicine, Division of Molecular Techniques, Wroclaw Medical University, Sklodowskiej-Curie 52, 50369, Wroclaw, Poland; agnieszka.chlopas-konowalek@umw.edu.pl
[3] Department of Forensic Medicine, Faculty of Medicine, Wroclaw Medical University, 4 J. Mikulicza-Radeckiego Street, 50345 Wroclaw, Poland; pawel.szpot@umw.edu.pl
[4] Department of Social Sciences and Infectious Diseases, Faculty of Medicine, Wroclaw University of Science and Technology, 27 Wybrzeze Wyspianskiego Street, 50370 Wroclaw, Poland
[5] Institute of Toxicology Research, 45 Kasztanowa Street, 55093 Borowa, Poland

* Correspondence: m.zawadzki@pwr.edu.pl
† These authors contributed equally to this work.

Figure S1. Structures of modafinil and IS (methylphenidate-*d*9).

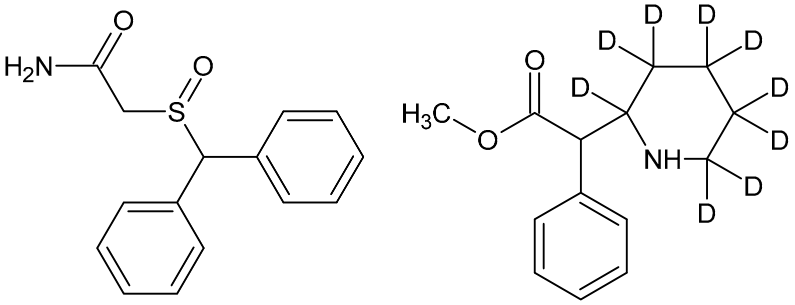

Modafinil Methylphenidate-*d*9

Figure S2. Tablets after removal from blisters.

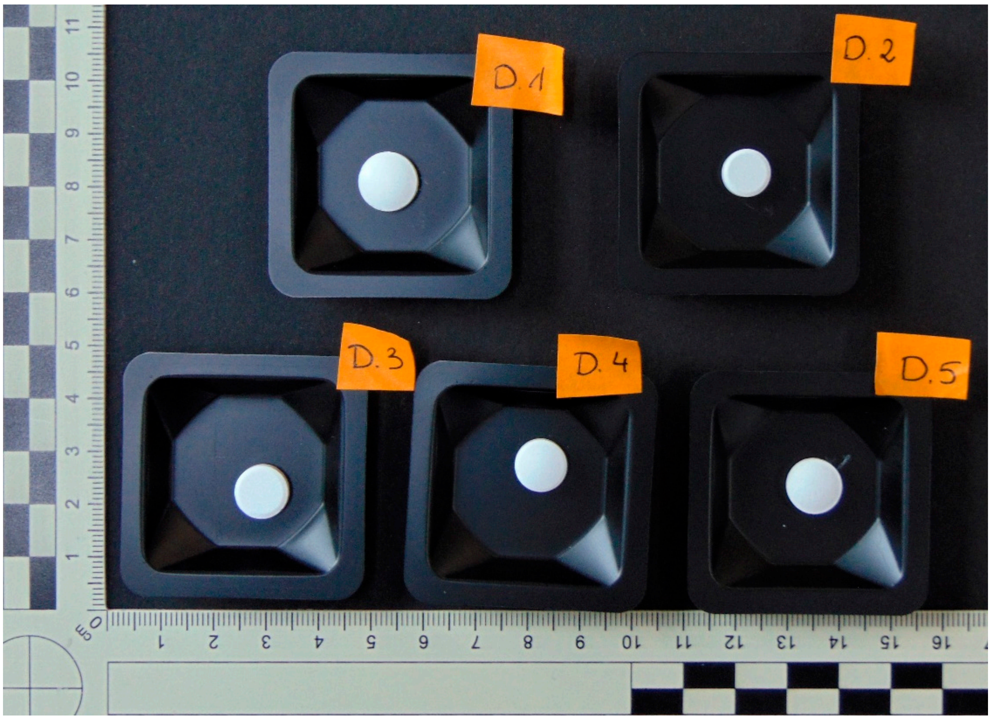

Figure S3. Proposed fragmentation of sodium adduct of modafinil (296 *m*/*z*).

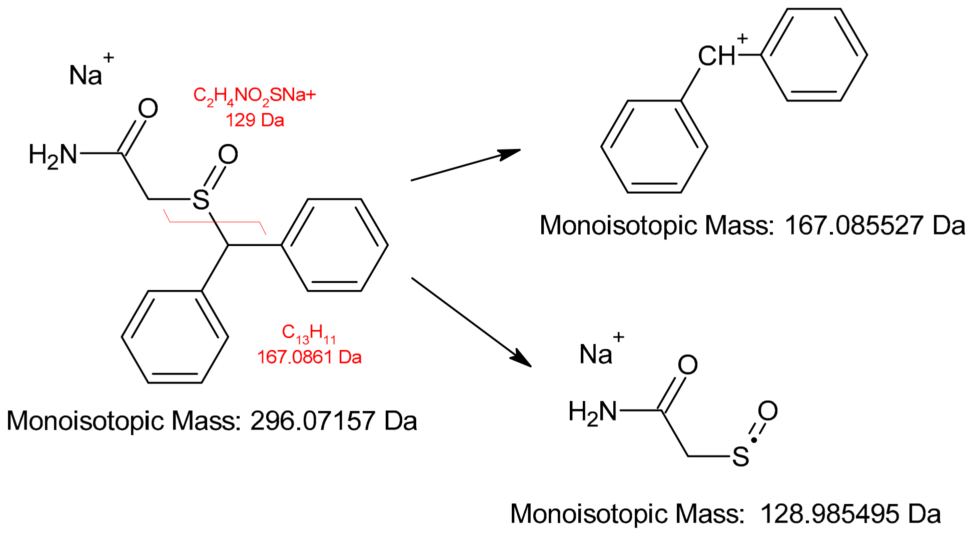

Figure S4. MRM of modafinil in blank methanolic solution sample (A), and stock solutions: 0.05 μg/mL (LOD) (B), 0.1 μg/mL (LLOQ) (C), 1.0 μg/mL (D), and 10.0 μg/mL (ULOQ) (E); MRM of IS methylphenidate-d_9 in 1.0 μg/mL (F).

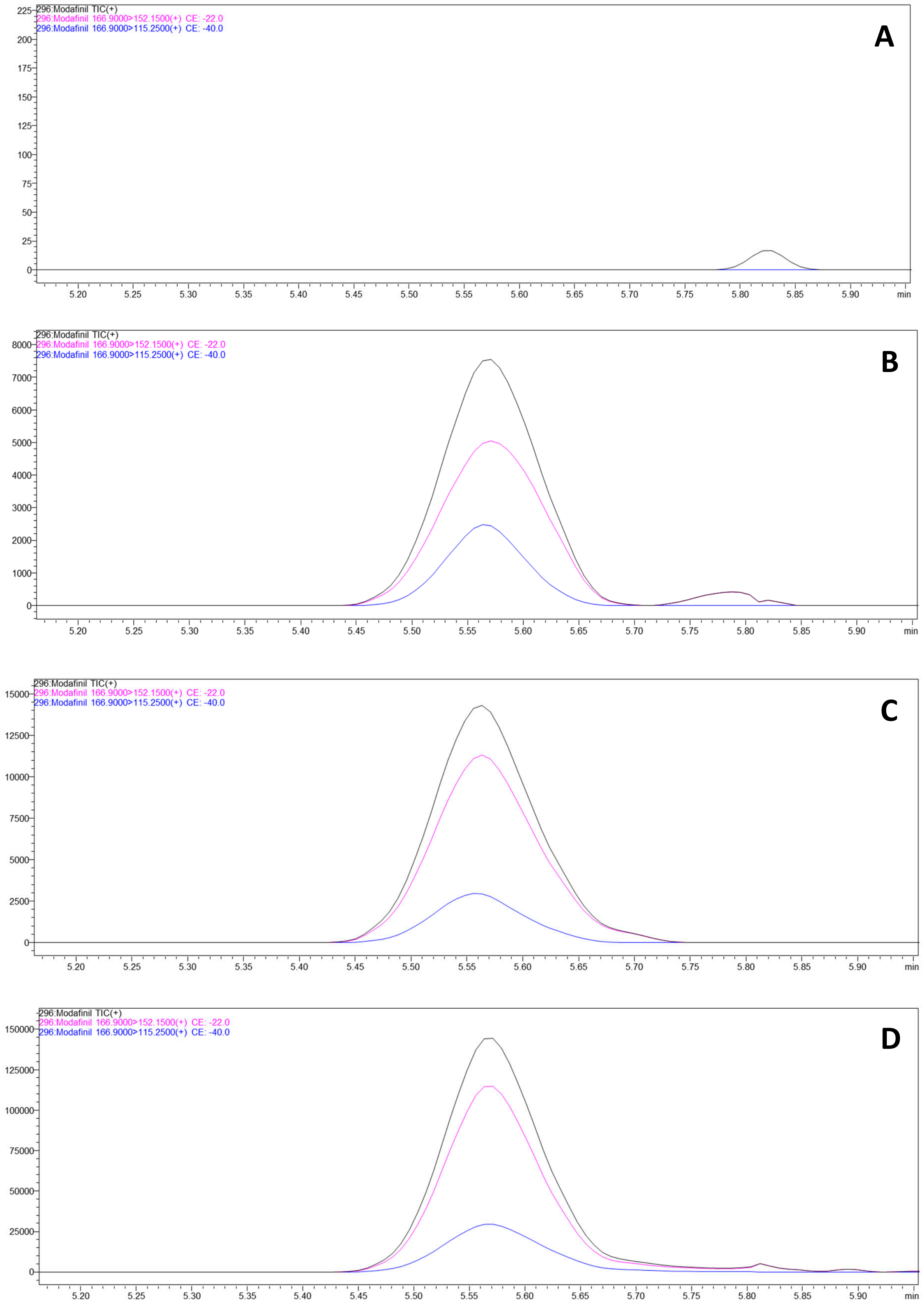

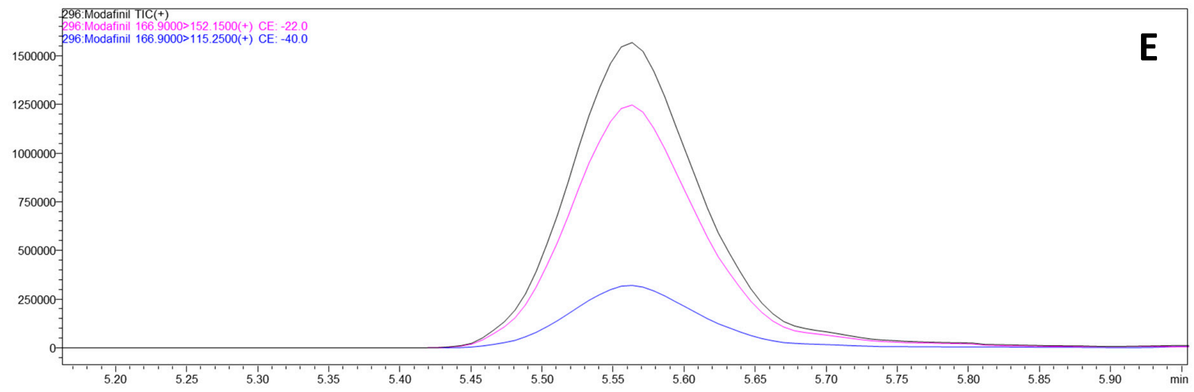

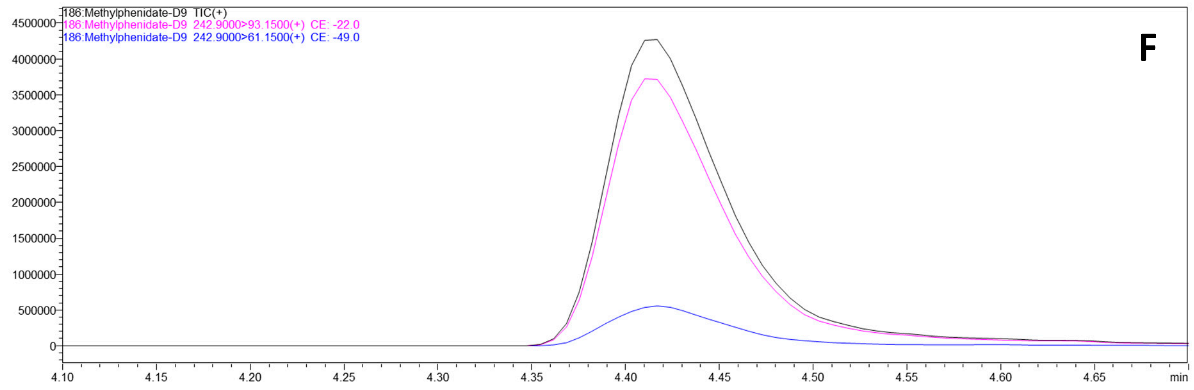

Table S1. Results of evidentiary sample method's validation; $n = 5$.

Validation results		
Intra-day precision [%]	0.2 μg/mL	3.3
	1.0 μg/mL	2.1
	8.0 μg/mL	2.2
Intra-day accuracy [%]	0.2 μg/mL	1.8
	1.0 μg/mL	4.2
	8.0 μg/mL	3.8
Iner-day precision [%]	0.2 μg/mL	4.0
	1.0 μg/mL	2.7
	8.0 μg/mL	1.9
Inter-day accuracy [%]	0.2 μg/mL	4.4
	1.0 μg/mL	3.9
	8.0 μg/mL	4.8

Table S2. Comparison of selected parameters of methods for determining modafinil in biological matrices with the liquid chromatography with MS/MS detection system; LLE- liquid-liquid extraction; SPE- solid-phse extraction; DI- direct injection; PP- protein precipitation; SALLE- salting-out assisted liquid-liquid extraction; MRM –multiple reaction monitoring ; RRT- relative retention time; SRM – single reaction monitoring; ESI- electro-spray ionisation; TIS - thermally and pneumatically assisted electrospray; AM- ante-mortem blood; PM- post-mortem blood;

Biological matrix	Extraction method	Column	Mobile phase	Gradient	Injection volume	IS	Precursor ion [*m/z*]	Target*/ reference ion [*m/z*]	Mode	Ionization source	Retention time	LLOQ [ng/mL]	Recovery [%]	Ref.
Blood, urine, vitreous humor, liver, kidney, brain	LLE	Kinetex XB-C18 (2.6 μm; 2.1 x 150 mm)<br>Column oven set on 40°C	A: 10 mM ammonium formate in water with 0.1% formic acid<br>B: acetonitrile with 0.1% formic acid	0 min, 5% B;<br>12 min, 98% B;<br>14 min, 98 % B;<br>15-20 min, 5% B	2 μL	Methylphenidate-d_9	166.9	152.1*/ 115.2	MRM; positive	ESI	5.57	1.0	111	This work
K_3EDTA plasma	SPE	Ascentis® C18 column (5 μm; 4.6 x 150 mm)<br>Column oven set on 35 ± 1°C	methanol:<br>2 mM ammonium acetate: acetic acid; 35:65:0.1% (*v*/*v*)	isocratic flow for 4.5 min	10 μL	Modafinil-d_5	274.2	167.0*	MRM; positive	TIS	2.79	30.8	86.7-91.7	89
Urine	LLE<br>DI	Sunfire C18 column (3.5 μm; 2.1 x 50 mm)<br>Column oven set on 20°C	A: 1 mM ammonium acetate in water with 0.001% acetic acid<br>B: 1 mM ammonium acetate in methanol with 0.001% acetic acid	0 min, 0% B;<br>2 min, 0% B;<br>6.5 min, 100% B;<br>7.5 min, 100 % B;<br>7.6-9.6 min, 0% B	50 μL	Mefruside	'[M+Na]⁺'	129*	SRM; positive	ESI	6.31	10 (LOD)	69 ± 20 (LLE)	90
Blood	LLE	Kinetex C18 (2.6 μm; 4.6 mm × 50 mm)<br>Column oven set on 60°C	A: 50 mM ammonium formate in water + formic acid (adjusted to pH 3.5)<br>B: acetonitrile + 0.1% formic acid	(-1)-0 min, 10% B;<br>0-0.25 min, 10% B;<br>0.25-3 min, 40% B;<br>3-4.25 min, 100% B;<br>4.25-5 min, 100% B	20 μL	Ephedrine-d_3	274.0	167.2*/152.2	MRM; positive	ESI	2.63	50	AM: (-2) ± 19; (-11) ± 23<br>PM: (-5) ± 33; (-13) ± 37	91
Urine	2-stage LLE preceded by	Supelco Discovery C18 column (5 μm; 2.1 mm × 150 mm)	A: water + 0.1% formic acid<br>B: acetonitrile + 0.1% formic acid	0 min, 15% B;<br>7 min, 60% B;<br>13 min, 100% B;<br>8 min, 100% B;<br>9 min, 100% B;	20 μL	17α-methyltestosterone and mefruside	274	165/128	SRM; positive	ESI	RRT: 0.75	300 (LOD)	No information	92

	hydrolysis	Column oven set on 40°C		9-13 min, 15% B										
		Halo® C18 columns (2.7 µm; 2.1 mm × 150 mm; and 2.7 µm; 2.1 mm × 100 mm)		0 min, 10% B; 3.5 min, 60% B; 5 min, 100% B; 7.2 min, 100%; 7.2-11.2 min, 10% B							'Halo 100 mm' RRT: 0.77 'Halo 150 mm' RRT: 0.79	300 (LOD)		
Urine	LLE	Inertsil C-18 column (3.0 µm; 4.6 x 50 mm) No information about column oven temperature	A: water with 1% formic acid B: acetonitrile	0-5 min, 15% B; 5-6 min, 60% B; 6-7 min, 100% B; 7-11 min, 15% B	No information	Mefruside	274	167	MRM; positive	ESI	5.1	100	from 101.2 ± 5.4 to 106.4 ± 4.9	93
Plasma	PP	Kinetex XB-C18 column (2.6 µm; 3.0 x 50 mm) Column oven set on 40°C	A: 2 mM ammonium formate in water with 0.2% formic acid B: 2 mM ammonium formate in acetonitrile with 0.2% formic acid	0–1 min, 2% B; 8 min, 65% B; 8.5, 65% B; 8.5-11 min, 2% B	20 µL	Trimipramine-d_3	296.0	128.9*/127.9	MRM; positive	ESI	5.85	5	from 103.0 ± 4.8 to 104.3 ± 2.0	94
Plasma	PP	UPLC BEH C18 column (1.7 µm; 2.1 x 50 mm) Column oven set on 40°C	A: acetonitrile B: water with 0.1% formic acid	0-0.2 min, 90% B; 1 min, 30% B; 2.5 min, 10% B; 2.8 min, 90% B; 4 min, 90% B	2 µL	Midazolam	274	167	MRM; positive	ESI	1.69	1	91.6-95.0	95
Plasma	PP	Phenomenex-C18 (5 µm; 4.0 x 50 mm) No information about column oven temperature	acetonitrile:methanol:0.1% formic acid 25:60:15 (*v/v*)	isocratic	No information	Modafinil-d_5	274.2	229.0	MRM; positive	ESI	0.91	2	97.26	96
Hair	Incubation in methanol	Kinetex C18 (2.6 µm; 3.0 x 50 mm) Column oven set on 40°C	A: 2 mM ammonium formate in water with 0.2% formic acid B: 2 mM ammonium formate in acetonitrile with 0.2% formic acid	0 min, 2% B; 8 min, 65% B; 8.5-10 min, 90% B; 10-14 min, 2% B	5 µL	Desipramine-d_3	274.2	167.0*/152.2	MRM; positive	ESI	5.7	200 [pg/mg]	87.9 ± 27.7	97
Urine	SALLE	Acquity BEH C18 column (1.7 µm; 2.1 x 100 mm) Column oven set on 45°C	A: water with 0.01% formic acid B: acetonitrile with 0.01% formic acid	0-0.6 min, 5% B; 3.8 min, 90% B; 4 min, 90% B; 4.1 min, 5% B; 5 min, 5% B	5 µL	7-propyltheophilline	274	167	MRM; positive	ESI	2.29	100 (LOD)	97.3	98

References to Table S1

[89] Bhartiyia, R., Mishra, S., Khuroo, A., Satyanarayana G.N.V.S. Liquid chromatography tandem mass spectrometry method for the estimation of modafinil in human plasma. *Word J Pharm Sci* 2014, *2(10)*, 1134-1415.

[90] Deventer, K., Pozo, O.J., Van Eenoo, P., Delbeke F.T. Qualitative detection of diuretics and acidic metabolites of other doping agents in human urine by high-performance liquid chromatography–tandem mass spectrometry. Comparison between liquid–liquid extraction and direct injection. *J Chromatogr A* 2009, *1216(31)*, 5819-5827.

[91] Di Rago, M., Pantatan, S., Hargreaves, M., Wong K., Mantinieks D., Kotsos A., Glowacki L., Drummer O.H., Gerostamoulos D. High Throughput Detection of 327 Drugs in Blood by LC-MS-MS with Automated Data Processing. *J Anal Toxicol* 2021, *45(2)*, 154-183.

[92] Mazzarino, M., de la Torre, X., Botre, F., Gray N., Cowan D. A rapid screening LC-MS/MS method based on conventional HPLC pumps for the analysis of low molecular weight xenobiotics: application to doping control analysis. *Drug Test Anal* 2010, *2(7)*, 311-322.

[93] Dubey, S., Ahi, S., Reddy, I.M., Kaur T., Beotra A., Jain S. A novel study of screening and confirmation of modafinil, adrafinil and their metabolite modafinilic acid under EI-GC-MS and ESI-LC-MS-MS ionization. *Indian J Pharmacol* 2009, *41(6)*, 278-283.

[94] Park, D., Choi, H., Jang, M., Chang H., Woo S., Yang W. Simultaneous determination of 18 psychoactive agents and 6 metabolites in plasma using LC-MS/MS and application to actual plasma samples from conscription candidates. *Forensic Sci Int* 2018, *228*, 283-290.

[95] He, Y., Ma, Y., Luo, L., Junying Ch., Congcong W., Meiling Z.Determination of modafinil in rat plasma by UPLC-MS/MS and a study of its pharmacokinetics and bioavailability. *Acta Chromatographica*, 2023, 35*(2)*, 187-192.

[96] Phanindra, A., Kumar, Y.S. Method development and validation of LC-ESI-MS/MS technique for the estimation of modafinil in human plasma; application to pharmacokinetics in healthy rabbits. *IJPSR* 2020, *11(4)*, 1837-1844.

[97] Sim, J., Kim, E., Yang, W., Woo S., In S. An LC-MS/MS method for the simultaneous determination of 15 antipsychotics and two metabolites in hair and its application to rat hair. *Forensic Sci Int* 2017, *274*, 91-98.

[98] Ventura, R., Roig, M., Montfort, N., Sáez P., Bergés R., Segura J. High-throughput and sensitive screening by ultra-performance liquid chromatography tandem mass spectrometry of diuretics and other doping agents. *Eur J Mass Spectrom (Chichester)* 2008, *14(3)*, 191-200.
